# Mothers’ views of their preschool child’s screen-viewing behaviour: a qualitative study

**DOI:** 10.1186/s12889-016-3440-z

**Published:** 2016-08-04

**Authors:** Georgina F. Bentley, Katrina M. Turner, Russell Jago

**Affiliations:** 1 Centre of Academic Primary Care, School of Social and Community Medicine, University of Bristol, Bristol, BS8 2PS UK; 2 Centre for Exercise, Nutrition & Health Sciences, School for Policy Studies, University of Bristol, Bristol, UK; 3 The National Institute for Health Research Collaboration for Leadership in Applied Health Research and Care West (NIHR CLAHRC West) at University Hospitals Bristol NHS Foundation Trust, Bristol, UK

**Keywords:** Preschool child, Parenting, Screen-viewing, Sedentary behaviour, Qualitative research

## Abstract

**Background:**

Research on screen-viewing in preschool children has predominantly focused on television viewing. The rapid development of mobile devices (e.g. tablets, smart phones and e-readers) and the increase in their use by preschool children means there is a need to understand how and why these devices are used by this age group. The aim of this study was to explore mothers’ views of their preschool children’s screen viewing behaviour (including mobile devices) and investigate how preschool children use different screen-viewing devices.

**Methods:**

One-to-one, semi-structured interviews with mothers of preschool children (aged between 2 and 4 years old). Mothers were recruited through preschools, nurseries, and mother and toddler groups located within four areas of varying socio-economic status within Bristol, UK. Data were analysed thematically using a framework approach.

**Results:**

Twenty-six mothers were interviewed. Mobile devices were regularly used as a form of screen-viewing for most children but were used on an ad hoc basis rather than being a habitual activity. The reasons and influences of mobile device use described by mothers were similar to that of television viewing. However, the portability of mobile devices meant that they were often used outside of the home as a distraction tool. Their multi-functionality meant that they could be used as a portable television, or for purposeful learning through educational games and applications. Some mothers showed concerns over mobile device use by their child, whilst others felt it was an important and useful educational tool. Although the majority of mothers felt they needed to set rules and restrictions for mobile device use, many mothers felt that they are also a necessary and unavoidable part of life.

**Conclusions:**

Mothers in this study suggested that mobile device use by preschool children is common. More research is needed to determine the impact of mobile device use in preschool children, how much time preschool children spend using mobile devices and which activities their use may be replacing.

## Background

For preschool children, screen-viewing mainly consists of television viewing but also includes computer use and, increasingly, the use of touchscreen mobile devices [1]. The current information on the associations between television viewing and health outcomes among young children is mixed. For instance, it remains unclear if television viewing is associated with overweight and obesity [2–9], and poorer or improved academic skill development [4, 10, 11] in young children. There is evidence of associations between television viewing and lower levels of physical activity [4, 12], shorter sleep duration [13–15], adverse dietary outcomes [4, 16], and poorer well-being outcomes [17] in young children. In general, however, more work is needed to understand these associations and particularly the extent to which associations could be explained by other factors such as such as parental sedentary habits [18]. Because screen-viewing is one of the few easily modifiable sedentary behaviours in preschool children, it is often targeted within interventions to reduce sedentary time.

In the UK, there are no specific government guidelines of daily screen-viewing time for young children, only that screen-viewing should be minimised [19]. The Australian [20] and Canadian [21] guidelines suggest that children between 2 and 5 years of age should have less than 1 h of screen-viewing time per day. The American guidelines have recently been revised to suggest that screen-time should be monitored and minimised without giving a specific recommended time limit [22].

Mobile devices, such as smart phones and tablets, are easily and intuitively used by very young children and provide an instant interactive element that appeals to both children and parents [1]. There are little data published that identify how much time preschool children engage with mobile devices in the UK. However, studies have reported that television viewing in preschool children is high. For example, a UK survey of 252 parents showed that two-thirds of 3 to 5 year olds were watching two or more hours of television per day [23]. A US survey with parents of children aged 0 to 8 years old reported that children’s access to mobile devices had increased from 52 % in 2011 to 75 % in 2013 [24]. In addition, children were watching less television per day (57 min in 2013 compared with 69 min in 2011) and spending more time using mobile devices (15 min in 2013 compared with 5 min in 2011), indicating that there has recently been a shift in screen-viewing behaviours and mobile devices are becoming more prominent in young children’s lives.

Parents greatly influence the screen-viewing behaviours of young children and act as ‘gatekeepers’ to the amount of screen-time and the type of devices children have access to [25]. This influence may come from parents role-modelling screen-viewing behaviours [23, 26–29], the media equipment parents provide their children [23, 28], parents’ attitudes to screen-viewing (e.g. belief it is a positive or negative behaviour) [28, 30], and the rules or limits parents set on screen-viewing [23, 31, 32].

Parents’ perspectives of their preschool child’s screen-viewing have been explored in qualitative studies [25, 33–36]. However, as most of these studies were conducted before the availability of mobile devices, they have tended to focus on only television viewing. These studies report that parents believe television viewing for preschool children is acceptable in moderation and if appropriately balanced with other activities [25, 33, 34, 36]. They suggest parents are happy with their children’s television viewing time [34–36]. Parents perceive television viewing to have many benefits, e.g. its educational influence [25, 33–36], its ability to calm children and help them relax [34–36], as a distraction tool to allow parents to do household tasks [25, 33–36], and as a behaviour management tool [35, 36]. However, some parents have concerns over the impact of television on their child, including the influence of inappropriate content and advertising [34, 36], adverse health outcomes [34, 36], its negative impact on behaviour and mood [25, 34, 36], impact on social skills [33, 34], and its addictive nature [25, 33]. Yet, it appears that these concerns over television viewing are not perceived to outweigh its benefits, with majority of parents in these studies reporting that they encouraged television viewing for their preschool child.

There is a paucity of research available about parents’ perspectives of newer technologies such as catch-up television (where selected programmes are automatically saved to watch later), internet television (which allows for programmes and video content to be viewed via the internet though video streaming technology), and mobile devices. A recent study by Carson et al. [33] looked at parents’ reactions to the Canadian sedentary behaviour guidelines, in which parents included mobile devices in their discussions. Parents felt that tablets and smartphones were useful distractions and educational tools, and thought their children found them very alluring. This indicates that parents view these devices in a similar way to television viewing. As television, computer and mobile device use are all termed as screen-viewing, it is important to understand if they are used by parents and children in the same way.

Considering the rise in accessibility to mobile devices and the continual advances in this type of technology, a more comprehensive understanding of how they are used by preschool children is needed. Furthermore, there is a need to break away from solely examining television viewing and begin to explore parents’ attitudes to these newer devices and investigate the factors that influence their use. This study aimed to explore mothers’ views of their preschool children’s screen viewing behaviour (including mobile devices) and investigate how different screen-viewing devices are used by preschool children.

## Methods

In-depth, semi-structured interviews were held with mothers of preschool children in order to explore their views of their children’s screen viewing behaviours. The study was approved by the University of Bristol, Faculty of Medicine and Dentistry ethics committee. GFB, a female PhD student who has experience and training in qualitative research, carried out the recruitment and data collection.

### Recruitment and sampling

To ensure socioeconomic and urban diversity in the sample, mothers were recruited within low, medium, and high-socio-economic status (SES) areas, within the City of Bristol, and one rural community (mid SES) located 13 km outside the city centre. SES was defined by thirds of the 2010 index of multiple deprivation (IMD) [http://data.gov.uk/dataset/index-of-multiple-deprivation] which is an area based measure of deprivation associated with the residential postcode.

Mothers were approached via children’s centres, day nurseries, preschools, and mother and toddler groups running in the four targeted areas. Fourteen centres were approached for recruitment. Of these, 8 allowed face-to-face recruitment with mothers and a further three allowed information to be given to mothers via centre staff. For locations allowing face-to-face recruitment, posters and leaflets about the study were displayed for at least one week before GFB attended to recruit mothers. During face-to-face recruitment, GFB explained the reasons for the study and asked mothers if they would be willing to take part in a one-to-one interview. Mothers were eligible to take part if they could speak English and had a child that was between 2 years old and about to commence formal schooling (between 4 and 5 years). Only mothers were recruited for this study, as mothers tend to be the main caregiver.

All eligible participants received a study information sheet before signing a consent form. Consent forms were received from 34 mothers (9 from high SES, 8 from mid SES, 7 from low SES and 10 from the rural area). Thirty-two of these women agreed to take part when approached during face-to-face recruitment, and two contacted the researcher having read the information sheet. GFB contacted the mothers by telephone to arrange the interview, at which point three mothers dropped out of the study (1 high SES, 2 rural). Interviews were arranged at a time and place that was convenient for the mother. Those who were unable or unwilling to meet face-to-face were offered the option of a telephone interview. A further four participants did not turn up to interview (2 low SES, 1 mid SES, 1 rural).

### Data collection

GFB conducted 26 interviews in total. Ten interviews were conducted in the mothers’ home, three within the location of recruitment (i.e. children’s centre), and 13 over the telephone. The mothers’ child was present within 9 of the interviews. Interviews lasted between 23 and 67 min (mean 45 min). Twenty-two interviews were held between April and June 2013. A further four were undertaken 11 months later in order to reach data saturation, i.e. no new themes emerged from the analysis. The time delay in conducting these four interviews was due to GFB being on maternity leave. Data collection and analysis were undertaken concurrently, so that themes from earlier data collection could inform the focus of later interviews and to determine when data saturation had been reached. A semi-structured topic guide was used to ensure consistency across the interviews. This paper focuses on questions around mothers’ views of their preschool child’s screen viewing behaviours (Table 1). A summary of the interviews were discussed with participants after each interview to ensure the researcher interpreted and captured their views as intended.

**Table 1:** Questions around mothers’ views of their preschool child’s screen viewing behaviours

1. Please can you tell me about yourself and your family? Probe: who lives with you, what ages are your children, do you and your partner work, what sorts of things do you do together as a family? 2. Can you talk me through a typical week for [child’s name]? 3. So thinking about your preschool child, tell me a bit about him/her - What type of play does your child have a preference for? (E.g. crafts, rough and tumble, imaginary play, watching TV or DVDs?) 4. What do you think about the TV as an activity for preschools children? Probe: What types of programmes they watch? How long they watch it for? Who with? What are the pros and cons of watching TV? 5. Does [child’s name] play with/use any electronic media devices such as computers, laptops, tablets, smartphones, or games consoles? 6. What are your views on these devices for preschool children? Probe: When/how do they use them? Who with? Any pros and cons?	

### Data analysis

Interviews were audio recorded, transcribed verbatim and anonymised. Field notes were made by GFB immediately following each interview. The data were analysed thematically [37]. This entailed reading and re-reading the interview transcripts to gain an understanding of the mothers’ views. GFB and KMT independently read a sample of transcripts to identify potential codes that could be applied to the data, which they then discussed and which subsequently provided the basis for a coding frame. Once an initial coding frame had been developed, GFB and KMT independently coded a sample of transcripts. Any discrepancies between their coding were discussed. These discussions led to the coding frame being revised, with new codes being added and some codes being removed or defined more clearly. Once the coding frame had been finalised, transcripts were imported into NVivo (version 10.0, QSR, Southport, UK) to allow electronic coding and retrieval of data. Once the data had been fully coded, data coded under specific codes were retrieved and overarching or central themes identified. To assist with the systematic interpretation of the data, an approach based on framework analysis [38] was then used. This entailed summarising data pertaining to specific codes in tables. Comparisons were then made within and across the interviews. The reporting of qualitative research in this paper is in accordance with the Standards for Reporting Qualitative Research (SRQR) checklist [39]. Quotes reproduced in this paper have been tagged with the interview number, whether the interviewee resided in the low, medium or high SES, or the mid SES rural location, and the sex and age of their preschool child.

## Results

Details of participants interviewed are provided in Table 2. Three of the mothers had two children of preschool age, in which case both children were discussed in the interview. Results are presented below under four main headings: how different devices were used; reasons for screen-viewing; mothers’ attitudes towards screen-viewing; influences of screen-viewing. Screen-viewing devices are amalgamated into three groups: television (includes televisions and DVDs); computer (includes personal computers (PC), laptop computers, and child version computers); mobile devices (includes tablets, smartphones, e-readers and child version tablets). Table 3 summarises the results and provides a comparison between these three groups of screen-viewing devices in relation to the four headings described above.

**Table 2:** Participant characteristics (*N* = 26)

	Number	Percent
Area of recruitment		
Low-SES	5	19.2
Mid-SES	7	26.9
High-SES	8	30.8
Rural Mid-SES	6	23.1
Mothers details		
Lone parent	4	15.4
Mothers employment		
None	16	61.5
Part-time	6	30.8
Full-time	2	7.7
Child details		
Child age (years)		
2	4	13.8
3	15	51.7
4	10	34.5
Child sex		
Female	11	37.9
Male	18	62.1
Only child		
Yes	6	23.1
No	20	76.9

**Table 3:** Summary of results and comparison of devices. An item is checked where it had been reported by at least one mother

	Television	Computer	Interactive media
How and why devices are used			
Set/regular time of day/week	✓		
Ad hoc times of day/week		✓	✓
Parent permission only	✓	✓	✓
Free access	✓		✓
Specific content chosen by parent	✓	✓	✓
Free play			✓
Independently	✓		✓
With family members	✓	✓	✓
Supervision needed		✓	
No supervision needed	✓		✓
Reasons for use			
For child to rest	✓		✓
Education		✓	✓
Babysitter	✓		✓
To calm child, prevent negative behaviour	✓		✓
Used as a punishment/reward	✓		✓
Family time	✓		
Computer skills / school ready		✓	✓
Distraction tool outside of the home			✓
Mothers attitude towards screen-viewing			
+ Educational	✓	✓	✓
+ Acceptable in moderation and balance	✓	✓	✓
+ Valuable behaviour management tool	✓		✓
+ Important skill development for child		✓	✓
-Concerns about unsuitable content	✓	✓	✓
-Concerns about addictive nature	✓		✓
-Concerns about change in behaviour	✓		✓
-Concerns over sedentary nature	✓		✓
-Concerns about solitary nature/social skill development			✓
-Feelings of needing to use screen viewing rather than wanting to	✓		✓
Influences of screen viewing			
Child’s engagement to device	✓	✓	✓
Siblings	✓	✓	✓
Fathers	✓	✓	✓
Mothers’ childhood experience	✓	✓	

### How different devices are used

Mothers mentioned a number of screen-viewing devices that their preschool children had regular access to. These included television, DVD player, laptop computer, PC, games console (e.g. Xbox or PlayStation), tablet, smartphone, and children’s computer or tablet (e.g. VTech). All but one mother owned at least one television. The mother who did not own a television said that her preschool child would watch catch-up television from a laptop. All mothers mentioned that their child had access to a smartphone. Their child’s use of a computer (PC or laptop) was less frequently mentioned.

Televisions, computers and mobile devices were used in different contexts. For example, television was usually watched within set time periods that often coincided with times that the mother needed to be doing something elsewhere, such as getting ready in the morning and cooking dinner in the evening. Mobile devices tended to be used more frequently than computers but not at set times.

Dedicated children’s channels appeared to be the main source of television programmes that children watched (some mentioned family shows at the weekend and DVDs), and channels with no adverts were preferred. Many mothers said that they used catch-up television or recorded programmes so that children could watch a particular programme when they wanted to. This also gave parents control so they could only watch the programmes they felt to be appropriate. Most children required their parents’ permission to watch television. However, some mothers allowed the television to be on in the background all day. Generally the television was watched by children independently or with a sibling, although some mothers mentioned watching with their child or as a family.

*“We tend to do a lot of iPlayer catch up. So we know what they’re watching,” P32, Mid SES, Girl, age 4*

*“I have a preference for CBeebies (BBC Children’s channel) so there’s no adverts because I can’t abide them.” P36, High SES, Boy, age 4*

Computer use was primarily facilitated by a parent or sibling and was seldom used in isolation. This was mainly because children lacked mouse control and computer skills at this age. Most commonly, the purpose of computer use was to play educational games and videos. Some mothers mentioned that their preschool child would also watch or participate in computer games with their older sibling just for fun.

The multi-modality of mobile devices meant they tended to be used in varying ways (i.e. watching programmes and films, playing games, educational applications (apps) and taking and looking at photos). Sometimes these devices were used with parent participation (usually a tablet), especially educational games and apps. More often, however, they were used by the child in isolation, either when the child requested it or when it had been given to the child by their parent. Access to mobile devices varied greatly. For instance, some mothers felt a need to ‘protect’ their child from their smart phone, while one mother mentioned that her children had free reign to her smart phone.

*“No I don’t let them have access, we don’t want them to be having access all the time (smartphone), I just don’t like it.” P35, High SES, 2 boys, ages 2 &3*

*“Yeah they have access to my iPhone all the time, when they get hold of that and I can’t find it anywhere…” P28, Mid SES Rural, Boy, age 4*

Some mothers mentioned providing their child with their own mobile device (e.g. iPad or iPod touch) from a young age (i.e. 18 months to 2 years) to provide their child with a form of entertainment and education.

### Reasons for screen-viewing

Mothers gave a range of reasons for why they allowed their child to screen-view. All the mothers said that screen-viewing was a good way for their child to rest, relax or have some quiet time. This screen-viewing mostly consisted of television viewing, although some mothers talked about giving their child a tablet or smartphone to play games or watch programmes on as a means of downtime. Screen-viewing was also encouraged by mothers when they felt their child getting too wound up or excited, to calm the child down and prevent disruptive behaviour. Again, television was the predominant device used. As mobile devices provides portable access to television programmes, these were also used for this reason.

*“She had an absolute meltdown about it and I just thought you need 20 min in her room. I put her in bed and I gave her my phone, and I let her watch Peppa Pig.” P32, Mid SES, Girl, age 4*

Screen-viewing was not the only activity mothers encouraged for down-time, some mothers (all from the high SES area) also mentioned they encouraged their child to read, do puzzles, crafts, listen to music, or play quietly on their own.

Mothers described how they refused their child screen-viewing time (predominantly television) as a punishment for bad behaviour and provided screen-viewing time (predominantly mobile devices) as a reward for good behaviour.

*“Yes it’s also a good tool to bribe them with, like if you do this, that and the other, you can get to use the iPad.” P13, Mid SES, Boy, age 4*

Often the reason for the child’s screen-viewing was to benefit the mother. For example, some mothers encouraged their child to screen-view when they wanted to do some household tasks, so they could sleep longer in the morning, or if they need to have a break from the child. Television was most commonly used in this instance, although mobile devices were also often mentioned.

*“I think sometimes it’s not just the need for them to physically stop but I kind of feel the need for them to mentally stop as well, if that makes sense… Um, and even if they are colouring they’re, you know, um, I get exhausted by being with them sometimes.” P36, High SES, Boy, age 4*

*“Like my older children have got iPads and, you know, he’s quite able to sit quietly and play on one of those as well, like games and things like that.” P53, Mid SES Rural, Boy, age 3*

The portable nature of mobile devices meant they could be used as a convenient and effective distraction for children in situations that required them to be patient and/or quiet outside of the home. For example, waiting for an appointment or when travelling in the car.

*“Erm just kind of if we were travelling or something like that she normally uses it erm the other day we went to the dentist and we took it with us because it’s quite useful to kind of you know while she’s waiting for us to have our teeth done kind of thing. They’re portable it’s so useful isn’t it?” P10, Low SES, Girl, age 3*

Television can be an opportunity for family time and closeness between family members. For example, mothers described cuddling with their child whilst watching television or a film, and described this as something that benefited both them and their child. This was not mentioned with other screen-viewing devices.

All mothers felt that screen-viewing provided a valuable educational opportunity. Children’s television programmes were thought to help children with language development, academic attainment and general knowledge. However, this appeared to be a consequential benefit of watching television and not the primary reason. Mothers described using computers to help their child’s learning (e.g. reading, letters, numbers, colours etc.) through games and videos. Games available on mobile devices were often seen as a fun, accessible and an easy way for mothers to help their children learn, and were mentioned by most mothers.

*“She knows her alphabet pretty well and I’m absolutely sure that’s from an app where she has to match up and it says the letter she matches up and erm….”P34, High SES, Girl, age 3*

Some mothers felt that developing computers and/or touchscreen skills were important for their child. This was mainly because they were aware that their child would be using these devices in school and wanted them to have a head start or feared that they would be behind if they did not have computer skills. Some mothers commented that computer use was an important component of modern life, and that children should be encouraged to understand and use it from an early age. One mother from the low SES area said that she did not feel that it was relevant for children to learn about technology until they were school age.

*“I think it’s important as well because obviously computers that’s … that’s life isn’t it, that’s modern. It’s no good him starting school not having used a computer and not having used a mouse because like even in Reception and Year 1 they’re using computers.” P39, Low SES, Boy, age 3*

*“I don’t know how useful it is for a pre-schooler, does that make sense? Whereas until he starts school and he starts however they learn they need to see it, try it, do it, get it right rather than sit down now.” P45, Low SES, Boy, age 3*

### Mothers’ attitudes towards screen-viewing

Often computer and mobile device use were described as more positive forms of screen-viewing than television viewing because they were less passive and required children to engage in activities. However, mothers also felt that mobile device use was a more solitary activity for children and therefore detrimental to social development, whereas television viewing was more inclusive and provided more opportunity for discussion.

Many mothers portrayed a sense of wonderment at their children’s ability to use mobile devices (mostly mothers from the high SES area). They found it rewarding to see their young child being competent at a skill. Some felt a sense of bemusement that their child used these devices so instinctively when they themselves did not feel so competent.

*“Technologically I think they’re amazing because they’re actually so much better than we are, things on the computer and I’m like I haven’t even taught you how to do that, she’s like no, no but I know if I press this button.” P14, High SES, Girl, age 4*

Many mothers were concerned about their perceived negative effects of screen-viewing, this was often regarding the content of the screen-viewing rather than length of screen time. For instance, some mothers (mostly mothers of boys) felt that some children’s television programmes and computer games may be a negative influence and encourage bad behaviour or violence. Many mothers talked about changes in their child’s behaviour when they screen-viewed, including their child being slower, having less energy or ‘zoning out’. This was predominantly relating to television viewing but also included mobile devices. Some mothers talked about the addictive nature of screen-viewing and were concerned that their child would form habits that would continue into older childhood and the teenage years. Only a few mothers mentioned concerns over the sedentary nature of screen-viewing. Typically, the educational value (especially with interactive devices) and the need for the parent to keep the child occupied, outweighed any concerns mothers had with screen-viewing. Some mothers did not like their child screen-viewing but felt a sense of resignation that they needed to use it as a tool to ‘babysit’ their child.

*“Well, we have in the house, we have sort of iPads and things like that, and I think too much of it is just… It zones them out, and you just can’t get any conversation out of them.”P28, Mid SES Rural, Boy, age 4*

*“I think it’s one of those that’s probably easier, it’s very, I don’t like them to watch too much TV, I don’t like the thought that they’re just sat here watching something but I think practically sometimes it’s just, well it is an easy option but I still, and I do do it at times but I don’t like doing it and I wouldn’t, I wouldn’t want to do it.” P13, Mid SES, Boy, age 4*

### Influences of screen-viewing

A child’s preference for screen-viewing appeared to influence the amount of screen-time allowed. Many mothers described the strong desire from their child to use mobile devices, and often parents felt they needed rules and restrictions in place to manage children’s demands for their use. These included hiding devices, only being able to use devices in their fathers’ presence and with his permission, pass-coding devices, and time limiting use. However, it appeared that some of these children may still spend long periods using mobile devices and what mothers felt was ‘too much’ screen-viewing varied. For instance, although one mother described restricting her child’s use of the family tablet, she also allowed the child to use the device for up to two hours in one session. Some mothers mentioned that their child would have tears and tantrums when a screen-viewing device was taken away, which mothers described as difficult to manage.

*Mother: “We’ve got them pass coded [iPad]. They can’t pick it up without our permission.”*

*Interviewer: “And how long would you let him use it for?”*

*Mother: “I guess not really much more than two hours”P55, Rural, Boy, age 4*

*“He does love it and he would ask for it and once he’s playing it’s difficult to encourage him not to. It would be difficult to get him off that onto another activity, difficult to upgrade from his favourite game.” P31, High SES, Boy, age 4*

Contrary to this, some mothers felt that their child did not need restrictions on screen-viewing in order to prevent extended periods of viewing. For instance, some mothers explained that their child did not have the attention span for extended periods of television viewing and could only watch 15 to 30 min of television before they moved onto something else. In addition, some mothers who allowed their child to have free access to mobile devices found that after an initial enthusiasm for the device (e.g. a couple of months) their interest in it wore off and they would naturally choose other activities to do, such as playing with toys over screen-viewing.

*“In fact he’s not used the iPad for a few months now. He’s not needed it, he’s been quite happy playing by himself with his toys. I’ve not thought about it and he’s not asked for it.” P41, Mid SES Rural, Boy, age 3*

Family members also influenced the preschool child’s screen-viewing behaviours. Some preschool children were described as taking an interest in what their older sibling was doing on a computer, watching or participating with their sibling. Some mothers said that their older child would teach their preschool child how to use the computer and play games with them. It also seemed that preschool children with older siblings were exposed to television programmes and computer games aimed at older children.

*“The big thing which again he’s probably quite young to be doing is Minecraft. Which is a horrible build game. So he’ll do that, but again that’s his older brother influencing him.” P55, Mid SES Rural, Boy, age 4*

Some mothers described their child’s father encouraging screen-viewing as a way of interacting with their child. A few mothers described this with some contention, as this behaviour went against their desire to restrict screen-viewing. These mothers said that their child’s father had different views towards screen-viewing (especially mobile devices) than themselves, where they felt fathers believed that there was no need for restriction because screen-viewing was harmless, fun, and important for skill development.

*“It’s a bone of contention because he can’t see the problem with it… he’s opinion is its just fun and it’s good and it does give you skills.” P34, High SES, Girl, age 3*

Mothers’ own childhood experiences appeared to influence how they felt about screen-viewing. For example, one mother grew up with very little technology and wanted her child to enjoy an equally active lifestyle. Whereas another mother grew up with the televsion on all the time and felt that this was a positive experience because it stopped it being a novelty and she carried this through with her son.

*“Oh we’ve always got the TV on, it’s pretty much always on because when I grew up the TV was always on, and it’s not a novelty at all… he won’t really sit down and watch it, it’s just always on.” P39, Low SES, Boy, age 3*

## Discussion

Television viewing was the main form of screen-viewing discussed by mothers in this study and appears to be the main form of screen-viewing for their preschool child. Analysis of the data, however, shows that preschool children frequently have access to mobile devices. How the preschool children use this form of screen-viewing often overlaps with how they use television viewing and computers. There also appeared to be some differences in their use. For instance, unlike computers, mobile devices allow for independent play which means mothers are able to use them in a similar way to the television (e.g. as a babysitter, independent quiet time). Mothers feel that mobile devices have a purposeful and interactive educational value and are often viewed positively. Mobile devices seemed to be used at ad hoc times when its use was required, rather than regular periods of the day, which was the case with television viewing, indicating its use may not be habitual at this age.

Qualitative studies have reported mothers’ reasons for their preschool children’s television viewing. For instance, as a way for children to rest [34–36], to use as an electronic babysitter [25, 33–36] and as a behaviour management tool [35, 36]. These reasons for television viewing were confirmed in this study but were also given for mobile devices use. In addition to these reasons, computers and mobile devices are used as an educational tool to facilitate learning by using educational games. Apps on mobile devices for preschool children are often promoted as educational. It appears to be commonplace for schools and educational and parenting websites to recommend educational apps to use with preschool children, thereby promoting their use as an educational tool (e.g. [40, 41]). The use of touch-screen devices as a tool for learning in preschool settings is reported to stimulate concentration and motivation for literacy activities, and provide opportunities for communication and interaction, independent learning and feelings of achievement in young children [42]. Within the home, a study of 106 3–5 year olds reported that the use of educational apps have been associated with higher letter sound and name writing skills but time on tablets was not associated with emergent literacy skills [43]. More research is needed on the potential role of touch-screen devices on learning in the early years. It is likely that the quality of the experience with touch-screen devices is more important for effective learning than duration using them [43].

A unique difference between mobile devices and other screen devices is their portable nature, which means that screen-viewing can take place outside of the home and can provide a means to distract the child in situations that require them to be patient (e.g. car journeys and medical appointments). Radesky et al., [44] observed mobile phone use by parents and children in fast-food restaurants, and reported that parents often gave their young children a mobile phone in order to distract or pacify them if they were becoming active or disruptive. It has been speculated that this reliance on mobile devices to counteract boredom may inhibit a child’s ability to self-regulate their behaviour [1], and the use of mobile devices in this way may inhibit important interaction opportunities [44], but currently there is little research to substantiate this.

Some mothers in this study, used mobile devices (especially smartphones) with caution and felt their allure to children difficult to manage. They also showed concerns about their accessibility, the child’s behaviour that mothers feel result from use of mobile devices, and the perceived possibility that screen-viewing might have a negative effect on their child. However, many of these mothers felt that mobile devices were now a necessary and unavoidable part of life and allowed their child to use them regularly with some reluctance. Similarly, Carson et al. [33], reported that Canadian mothers of preschool children had some reservations about using screen-viewing as a babysitter but could not think of any viable alternatives. In contrast, some mothers in this study do not show concerns over screen-viewing for their child and support its use, this includes television viewing, computer use and mobile device use. For television viewing it seems that this is because there is no perceived harm in watching it, whereas mobile devices and computer use is encouraged for educational purposes. A qualitative study in six European countries concluded that parents do not have concerns over their child’s television viewing or computer use, however their views on mobile devices were not reported [34]. Nevertheless, most (but not all) mothers in this study felt the need for rules and restrictions to manage their child’s screen-viewing. This is consistent with findings from a quantitative study in Canada that reported 81 % of parents of 3 year olds had household rules for screen time [32].

It is clear that mobile devices provide an intuitive, responsive and interactive component that other screen viewing devices cannot provide. Research has not yet ascertained as to whether mobile device use can be defined in the same way as television viewing, and if perhaps, its use should be evaluated as a separate behaviour. However, it is important to note that, although these screen-viewing devices vary in the way they are used, they all may still be considered a sedentary activity. As sedentary behaviour in young children is associated with negative health outcomes, it is important that their use is still minimised. This study shows that mothers regularly provide mobile devices for their preschool children and highlights the need for further research to determine the impact of its use in young children. It is not known how much time preschool children spend with these devices and further research is need to establish whether mobile device use is replacing an alternative sedentary activity (such as television viewing or reading books) or other (physically active) activities. In addition, this study indicates differences between mothers and fathers views on screen-viewing, and that fathers influence their preschool child’s screen-viewing behaviour. Therefore, further research should be carried out to explore fathers’ views and influences of screen-viewing in preschool children.

The results of this study indicate that health policy and interventions aimed at reducing sedentary behaviour in young children need to be sensitive to the needs and priorities of parents and take into the account the reliance that parents have on screen-viewing devices. A qualitative study by Evans et al., reported that parents felt reducing television viewing in their 6 to 7 year old children would cause conflict in the home and require resources (e.g. financial and time) that they were unsure they could provide,[45]. This highlights the potential stress parents may be placed under when asked to change their child’s screen-viewing behaviour.

### Strengths and limitations

The interviews were held with a diverse sample of mothers, i.e. individuals from different SES areas, which included both urban and rural areas, working and non-working mothers, and lone-parents. However, the extent to which the findings can be generalised will be limited by the fact that the majority of the interviewees were white British. As mothers were aware of the nature of the research (i.e. investigating mothers’ views of their preschool child’s physical activity and sedentary behaviour) they may have been inclined to give socially desirable responses. As we do not have information on the age of the mothers in this sample, we are unable to comment on the generation from which the mothers belong or the extent to which their views are affected by the nature of their own screen-viewing as a child. Another limitation is that this study is based on interviews rather than observations, and so reports mothers’ perceptions of their child’s behaviour, rather than directly recording it.

## Conclusion

Mobile devices use is common in preschool children, although it does not appear to be a habitual behaviour. Their multi-functionality means that they may be used for independent play in the same way as television viewing (e.g. as a babysitter, independent quiet time), or more purposefully for learning through games and through computer skill development. A unique characteristic of mobile devices is its portability, meaning that screen-viewing can take place outside of the home. Although mothers expressed concerns over mobile devices use, they were generally viewed it more favourably than television viewing. The majority of parents had rules and restrictions to limit their child’s use of mobile devices. However, the strong allure of mobile devices to preschool children makes restricting their use problematic.

## Abbreviations

AAP, American Academy of Paediatrics; App, mobile devices application; IMD, index of multiple deprivation; PC, personal computer; SES, socioeconomic status, UK, United Kingdom
